# “MicroMED” Optical Particle Counter: From Design to Flight Model †

**DOI:** 10.3390/s20030611

**Published:** 2020-01-22

**Authors:** Diego Scaccabarozzi, Bortolino Saggin, Riccardo Somaschini, Marianna Magni, Pietro Valnegri, Francesca Esposito, Cesare Molfese, Fabio Cozzolino, Giuseppe Mongelluzzo

**Affiliations:** 1 Department of Mechanical Engineering, Politecnico di Milano, 23900 Lecco, Italy; bortolino.saggin@polimi.it (B.S.); riccardo.somaschini@polimi.it (R.S.); marianna.magni@polimi.it (M.M.); pietro.valnegri@polimi.it (P.V.); 2 INAF-Astronomical Observatory of Capodimonte, Salita Moiariello 16, 80131 Naples, Italy; francesca.esposito@inaf.it (F.E.); cesare.molfese@inaf.it (C.M.); fabio.cozzolino@inaf.it (F.C.); giuseppe.mongelluzzo@inaf.it (G.M.)

**Keywords:** MicroMED, thermo-mechanical design, Dust Suite, ExoMars 2020, flight model, qualification testing

## Abstract

MicroMED (Micro Martian Environmental Dust Systematic Analyzer (MEDUSA)) instrument was selected for the ExoMars 2020 mission to study the airborne dust on the red planet through in situ measurements of the size distribution and concentration. This characterization has never been done before and would have a strong impact on the understanding of Martian climate and Aeolian processes on Mars. The MicroMED is an optical particle counter that exploits the measured intensity of light scattered by dust particles when crossing a collimated laser beam. The measurement technique is well established for laboratory and ground applications but in order to be mounted on the Dust Suite payload within the framework of ExoMars 2020 mission, the instrument must be compatible with harsh mechanical and thermal environments and the tight mass budget of the mission payload. This work summarizes the thermo-mechanical design of the instrument, the manufacturing of the flight model and its successful qualification in expected thermal and mechanical environments.

## 1. Introduction

MicroMED (Micro Martian Environmental Dust systematic analyzer) was developed by a consortium of Italian research institutes and Spain’s space agency on the basis of the heritage of MEDUSA (Martian Environmental Dust Systematic Analyzer), a particle counter designed for the Humboldt payload [1,2]. MicroMED’s goal is measuring the abundance and size distribution of atmospheric dust close to the surface of the red planet, where dust lifting takes place. Such kinds of measurements have not been performed before on Mars, even though dust distribution in the Martian atmosphere has gathered so much attention in the scientific community, as reported in many studies exploiting indirect measurement methods applied to data collected by various experiments [3,4,5,6,7,8]. The expected impact of the MicroMED sensor will be the improvement of knowledge and understanding of the planet’s climate, since it is well recognized that airborne dust and regional or global dust storms cause changes in the atmospheric thermal structure of Mars.

MicroMED will be mounted on the Dust Suite of the surface platform onboard the ExoMars 2020 ESA and Roscosmos mission. The Dust Suite payload is composed of five sensors developed to image the landing site, monitor the long-term climate, and perform investigation and studies of the Aeolian processes on Mars. The instrument working principle is typical of optical particle counters (i.e., an optical system measures the light scattered by the dust particles passing through a collimated laser blade); from the light profile levels and duration information about the dust grain size and speed can be obtained.

In order to be approved for launch with the mission, the instrument must prove compatibility with harsh mechanical and thermal environments, due mainly to the launcher and the descent module dynamic excitations foreseen in the mission profile and predicted temperature scenarios on Mars. Moreover, a limited mass allocation of 500 g including electronics was made available for this instrument. The feasibility design of the MicroMED has already been reported in previous studies [9,10,11,12,13], evidencing the possibility from analysis to fulfill the design requirements of the mission with the available resources.

In this work, the detailed thermo-mechanical final design of the instrument is reported along with the experimental activity carried out in expected mechanical and thermal environmental conditions to validate the developed numerical models of the instrument. Moreover, the tests required to qualify the MicroMED Flight Model prior to integration on the ExoMars landing platform are presented.

## 2. Methods

### 2.1. Instrument Layout

**Figure 1:**
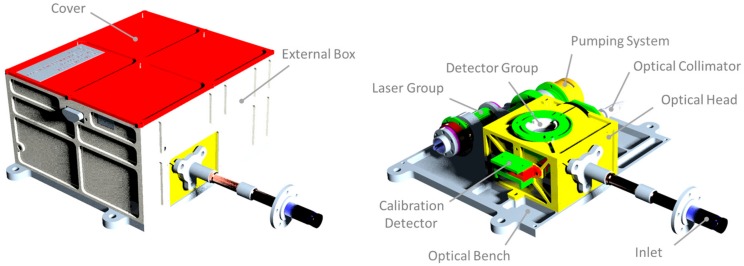
View of the MicroMED (Micro Martian Environmental Dust systematic analyzer) Flight Model layout three-dimensional (3D) CAD model.

MicroMED layout is made by different subassemblies. View of the main parts and components is provided in Figure 1.

The optical head (OH) is the main part of the instrument that holds science and reference detectors, the optical collimator (OC), the instrument fluid dynamic subsystem (inlet and outlet parts), and differential pressure sensors and temperature sensors. The selected detectors are Si PIN photodiodes (S5106 type @Hamamatsu), providing a sensing area of 5 × 5 mm^2^ and compatibility with a low pressure environment. One of the two detectors is used to measure scattered light from analyzed particles, whereas the second one (used for calibration) tracks potential degradations of the laser and of the main detector as the consequence of aging and radiations. More details about the instrument’s optical layout and performances are found in [9].

The selected pressure sensor is a differential MEMS pressure from Amphenol Nova Sensors (NPH-8-002.5-GH MEMS type). Its objective is to measure the differential pressure between the OH and the environment since, combining the measured pressure difference with the pressure loss characteristic curve of the instrument fluidic system, the measurement of the flowrate can be performed, allowing the assessment of the atmospheric dust density (i.e., the number of particles per unit of volume in the atmosphere). The pressure sensor is an off-shelf component, designed to operate in Earth’s environment, but to be adopted for the mission, it underwent full performance characterization in the expected temperature range, in low pressure conditions, and with the expected dose of radiation during the mission. The performed characterization proved the suitability of the selected sensor for the intended application [14].

The pumping system (PS) is based on a space-designed rotary vane pump with an embedded brushless motor; it must provide the differential pressure required for the atmospheric gas and particles to flow through instrument sampling volume. Design feasibility of the PS concept was already reported by Scaccabarozzi and colleagues [15], but its full characterization in the representative environment was performed prior to integration in the MicroMED Flight Model. A testing campaign was performed with a specifically designed instrument to measure the flowrate of gasses in low pressure conditions [16]. The obtained calibration curves of the PS highlighted proved compatibility with the required fluid dynamic performances predicted by CFD analysis [17].

The OC concentrates laser light, conveyed by an optic fiber in the center of the OH, generating a sampling volume of 1 × 1 × 0.24 mm^3^ where the scattering of the particles occurs. The scattered light is focused by a parabolic mirror on the science photodiode. Thanks to the optical and CFD design, MicroMED is able to directly measure both the size distribution and concentration of dust grains suspended in the Martian atmosphere, exploring the particle size range from 0.4 μm to 20 μm.

Pt1000 temperature sensors are installed on the sensitive components of the MicroMED (i.e., LG, PS, OH) and on the electronics as well, in order to monitor the temperature during operative and non-operative phases and assure safety of the installed components. Moreover, one temperature sensor is located on the tip of the instrument’ inlet, to provide an estimate of the Martian atmosphere temperature. The latter is expected to be different from the MicroMED interface one, and its knowledge is fundamental to accurately evaluate and control the flowrate within the instrument’s fluid dynamic circuit. The control of flowrate is needed, especially to achieve the required dust speed on the sampling volume where science measurements are performed.

The main instrument components are mounted on the optical bench (OB), which is the interface with the Dust Suite platform. Finally, the external box (EB) holds the electronics group, mechanically shielding the instrument’s components from external dust or contamination. The electronics allows conditioning and controlling of the subsystems and sensors and acquisition of the instrument housekeeping. The electronics is mounted on the instrument top and the EB provides an EMI shielding.

### 2.2. Thermo-Mechanical Design Requirements

Thermo-mechanical design requirements have been derived from the Surface Platform Information Package document [18]. The expected mechanical environment requires instrument survival with sweep sine and random excitations, whose profiles are summarized in Table 1 and Table 2.

Based on the launch and landing levels and gained experience from previous studies [19,20,21,22], the dynamic requirement was first set to a natural frequency larger than 150 Hz and a quasi-static loading to be used for the design phase of 1000 m/s^2^. Moreover, size and mass allocations for MicroMED development were limited to a maximum envelope of 148 × 200 × 70 mm^3^ and 500 g mass, respectively.

**Figure 2:**
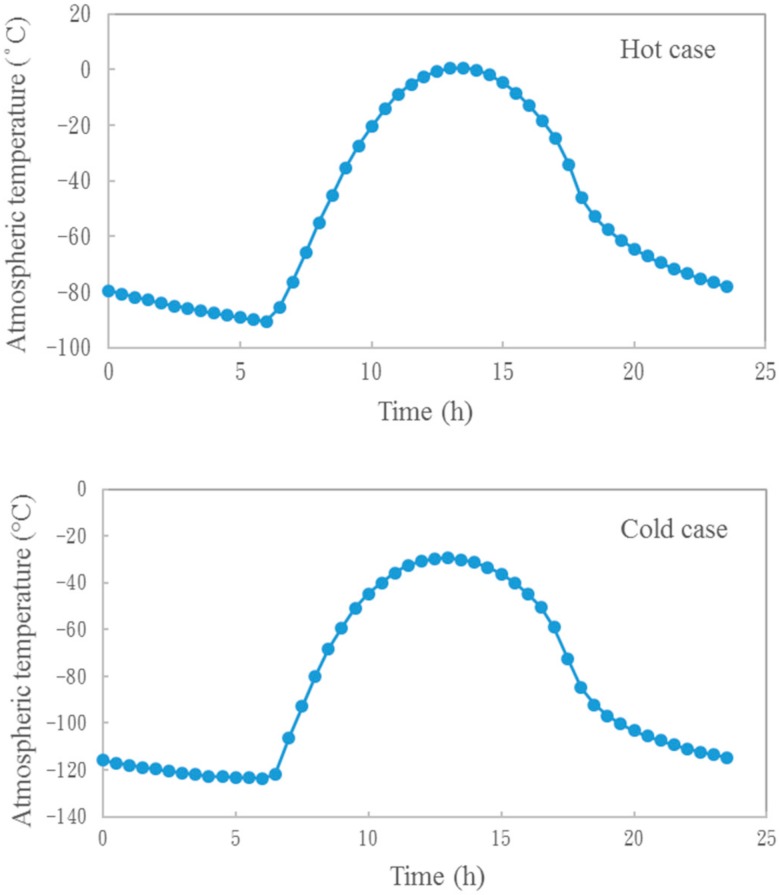
Hot (top) and cold (bottom) cases predict atmospheric temperatures for Mars.

Temperatures at the Dust Suite Platform interface are between −50° C to 40 °C or between −20 °C and 40 °C for the storage and working phases, respectively. It should be noted that the MicroMED requires sampling of the Martian atmosphere through the inlet. Thus, the thermal design must account for the Martian atmosphere temperature variation and it has to be verified that the instrument does not cause large heat dissipation to the environment through its sampling inlet. Moreover, in order to ease the unit thermal control, the MicroMED is covered with thermal insulator (about 9 cm thickness) with protrusion of the inlet in the Martian atmosphere of at least 1 cm. The expected Martian temperature profiles in hot and cold working scenarios [18] are shown in Figure 2.

The temperature trends shown in Figure 2 are used in thermal analysis to evaluate temperatures on sensitive elements of the MicroMED.

### 2.3. Flight Model Thermo-Mechanical Design

In order to complete the mechanical design of the MicroMED Flight Model, the finite element (FE) approach is used. Feasibility design of the MicroMED was already assessed in a previous study [9], showing the fulfillment of the requirement on the mass budget by means of mechanics based on ribbed geometry, which was optimized both for the OB and EB. Hereafter, the main results of the detailed design are presented.

Different FE models of the MicroMED assembly were developed and related analyses were performed to verify the mechanical resistance vs. quasi-static loading and instrument dynamic behavior. The assembly FE model comprises 59,885 tetrahedral and triangular elements and 17,274 nodes. The main subassemblies (i.e., OB, PS, OH, EB) and other components (inlet, outlet, and detectors) were modeled as lumped masses. This was mandatory in order to manage the FE model without jeopardizing the results’ accuracy. The lumped masses were connected to their supporting structures by weighted links and the OB was fixed simulating the bolt’s connection. The main material for the mechanical structures was aluminum alloy Al7075-T6, whereas the inlet tip, which is directly immersed in the Martian atmosphere, was designed with Vespel SP22. The latter is a well-known plastic material with space heritage. Details about the materials used in the FE model are given in Table 3.

In order to evaluate mechanical resistance in the expected temperature environment, a FE model was developed to investigate instrument behavior for the worst-case temperature scenario (i.e., mounting at room temperature and achievement minimum non-operational temperature). The model comprises the same features previously described with the addition of an aluminum plate 6061, to simulate Dust Suite mounting interface.

**Figure 3:**
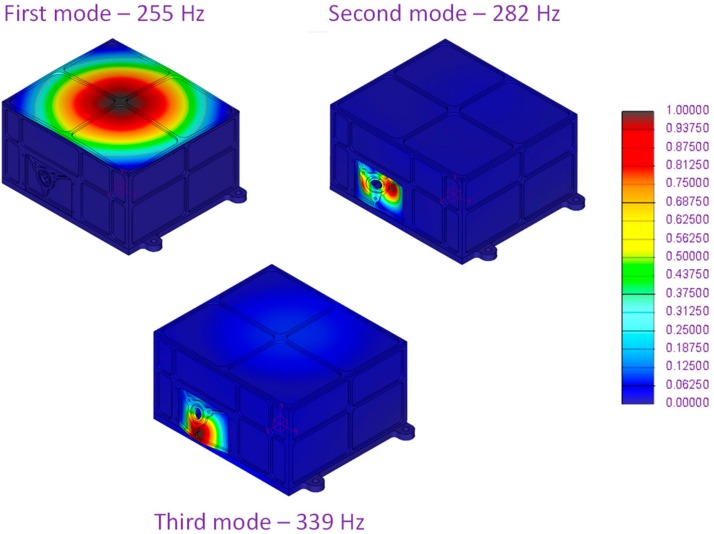
Computed non-dimensional displacement from modal analyses.

**Figure 4:**
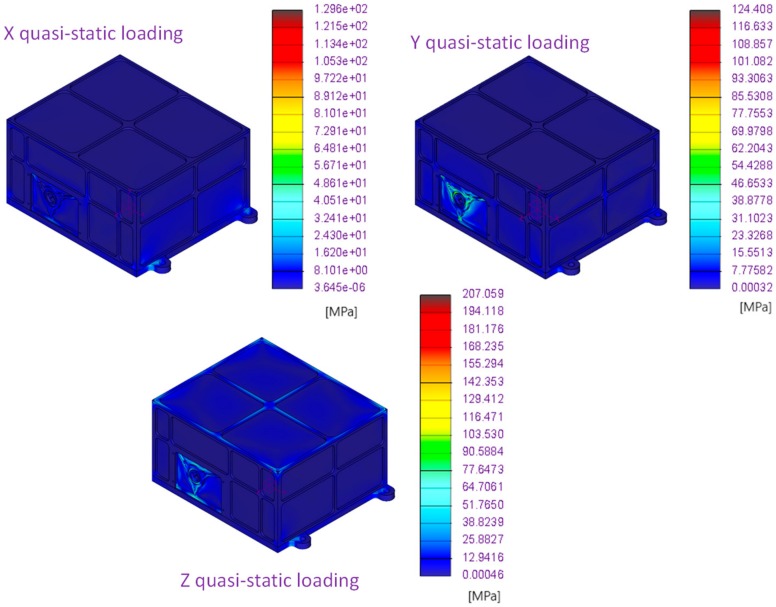
MicroMED Flight Model, quasi-static analyses results.

**Figure 5:**
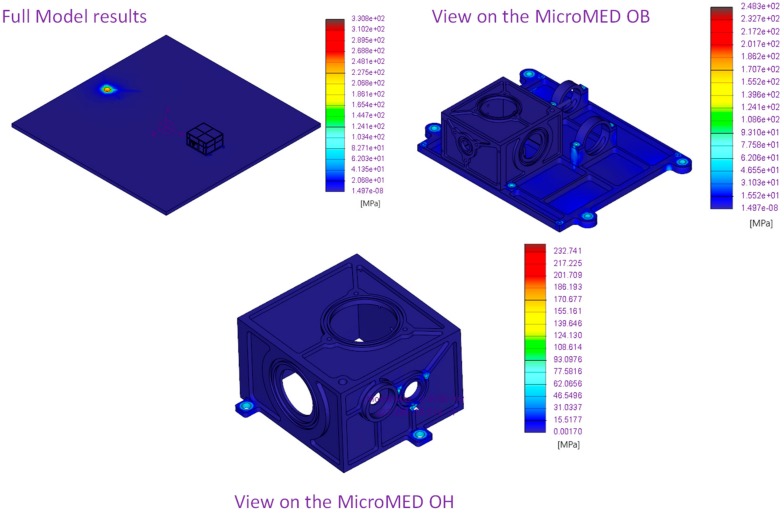
MicroMED Flight Model, thermo-elastic analyses results: computed Von Mises stresses on the MicroMED optical head (OH) and optical bench (OB), non-operative case, environmental temperature at −50 °C.

Results of first three modes of vibration and Von Mises stress along three main axes with quasi-static loading are provided in Figure 3 and Figure 4, respectively. Computed temperature distribution on the MicroMED for the thermo-elastic analysis is shown in Figure 5.

Table 4 summarizes the results of the mechanical resistance with quasi-static loading and thermo-elastic analyses, providing the computed margin of safety (MOS), which according to the ESA standard [23] is defined as:

(1) $$MOS=1-\frac{\sigma_{c}}{\frac{\sigma_{lim}}{\eta_{lim}}}$$

where *σ_c_* is the computed Von Mises stress, *σ_lim_* is the maximum admissible stress, and *η_lim_* is the related safety factor with respect to the limit condition. As for Equation (1), the MOS shall be positive in order to validate the mechanical design.

### 2.4. MicroMED Thermal Model

**Figure 6:**
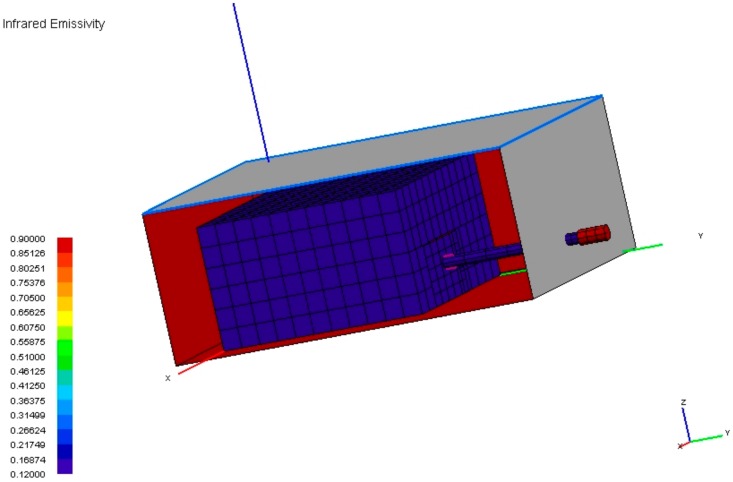
MicroMED thermal model, emissivity distribution over the geometrical model.

A thermal model of the MicroMED was developed to determine the temperature ranges in various conditions for the main instrument parts. The unit was therefore modeled with shells representing the main structures, electronics board, and power sources (i.e., the PS and LG subassemblies). A convective link with the only exposed surface (i.e., the instrument inlet) was set with a convective coefficient of 0.2 W/(m^2^K) and 2 W/(m^2^K) for the hot and cold cases, respectively. Infrared emissivity of the developed model is shown in Figure 6. Thermo-optical properties used for the surfaces of the model are summarized in Table 5.

As already explained, the thermal control architecture is based on a thermally conductive mounting on the Dust Suite platform as the temperature range specified at the interface compatible with the thermal requirements of all MicroMED components. The heat generated internally is therefore mostly damped through the mounting interface. Power dissipated by electronics ranges between 1.7 W and 2 W in operative hot and cold cases, respectively. The ON state duration for the electronics is about 300 s (time for each cycle) for the hot case and 126 s for the cold case. Power budgets for the PS and for the LG in both the operative cold and hot cases are 2.5 W and 0.4 W, respectively. Cycle time for both the LG and PS is set to 100 s.

**Figure 7:**
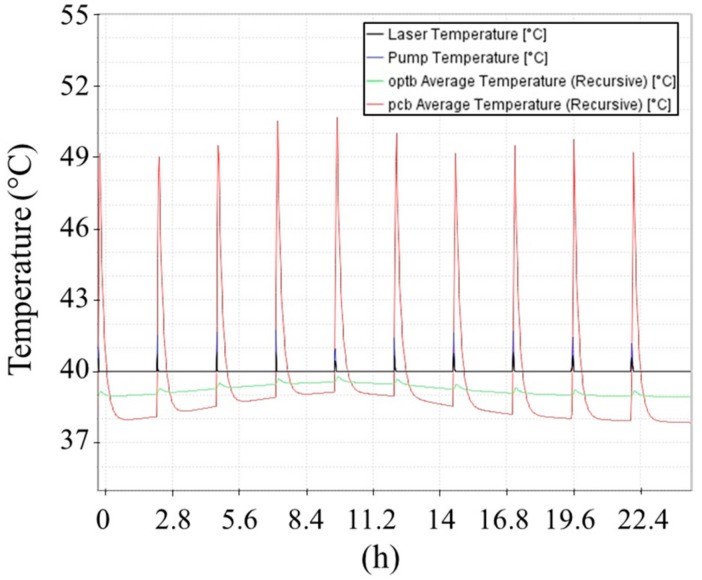
Hot operational case: temperature of critical components during second solar day of Mars operations.

**Figure 8:**
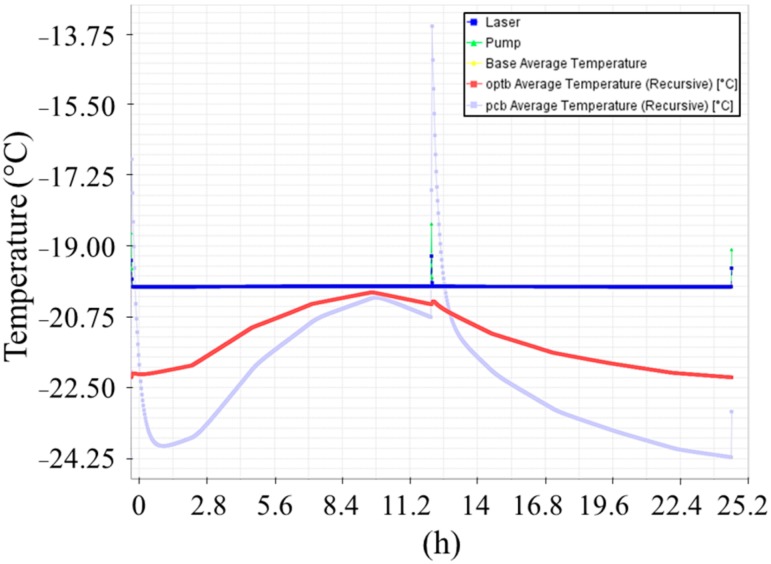
Cold operational case: daily cycle after two operational days.

The applied conditions for environmental temperatures are shown in Figure 2. The results of the thermal simulations are provided in Figure 7 and Figure 8.

Computed power exchanges at the Dust Suite interface are 1.7 W and 2.9 W in hot and cold operative scenarios, respectively.

### 2.5. Discussion

Performed FE analyses allowed verification of the MicroMED resistance within expected mechanical and thermal environments. It should be noted that the MOS for static and thermo-elastic simulations is larger than the minimum required. The modal analyses showed that MicroMED dynamic behavior is compliant to design requirements, highlighting first resonance about 60% over the minimum admissible value by design, providing a large safety margin with respect to the expected increased compliance due to real mounting and fixation.

The thermal analyses showed compatibility between the requirements for MicroMED’s components and the predicted temperatures. The laser was the only part whose predicted operating temperature exceeded the manufacturer-specified maximum temperature; this result was unavoidable because the max operating temperature for the mounting interface is coincident with the laser operating one. Nevertheless, the predicted laser temperature increase was below 1 °C, a value that is considered not relevant for the reduction of the component lifetime. Moreover, refined thermal analyses with a more detailed geometrical model of the instrument provide evidence that temperature uniformity is generally achieved on the OH, with maximum temperature difference of about 0.3 °C for both operational cases.

Finally, the temperature of the MicroMED inlet tip is always different from the atmospheric one because of both the low convective exchange with the Martian atmosphere and the limited thermal insulation between the sensor and instrument body. Provided that the inlet tip temperature is the closest approximation of the atmospheric one, a correction procedure shall be developed in case the atmospheric temperature cannot be retrieved from other instruments of the atmospheric package sampling volume.

## 3. Manufacturing and Integration

MicroMED main components were manufactured and checked before instrument integration. Main tasks were focused on geometry, mass, design tolerances, and interference checking. After manufacturing, coating (SurTec 650 Tri-chrome) was applied on all the mechanical parts. This finishing is required for any flight mechanics to avoid passivation and stress corrosion of the sensitive 7075T6 alloy.

**Figure 9:**
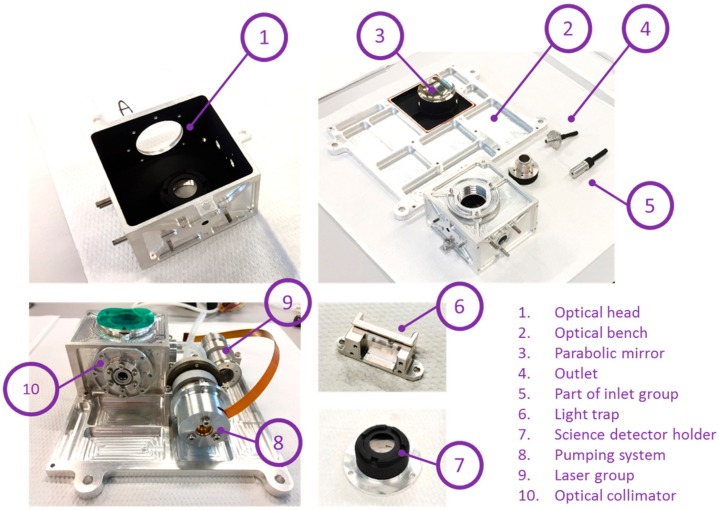
View of the manufactured components, preassembling activities.

In Figure 9 some examples of manufactured components and pre-assembled elements are shown.

Developed FE models were validated by means of experimental modal analyses on the manufactured main components.

In order to perform qualification testing on the MicroMED Flight Model, a protection box was designed and manufactured. The box provides the mechanical and thermal interfaces for the tested item, assuring fulfillment of the planetary protection requirements, even when the MicroMED must be transported and mounted on the Dust Suite interface. Thus, the MicroMED was integrated in a clean environment at the INAF-OAC (Astronomical Observatory of Capodimonte) clean room and mounted inside the box prior to qualification. In order to measure acceleration levels and temperature during the mechanical and thermal tests, different sensors were applied:

1. three piezoelectric accelerometers (Endevco 27A11 model, Brüel & Kjaer, nominal sensitivity 10 mV/g) placed on the top of the MicroMED Flight Model to sense acceleration along three testing directions (X, Y, and Z); and
2. four temperature sensors (Pt 1000, class A, IEC 751-95) were used: one was mounted on the MicroMED mounting interface (identified as the temperature reference point (TRP)), and the other three were positioned on the cover, on the OH, and on the inlet in order to have temperature distribution over the instrument during thermo-vacuum phases.

**Figure 10:**
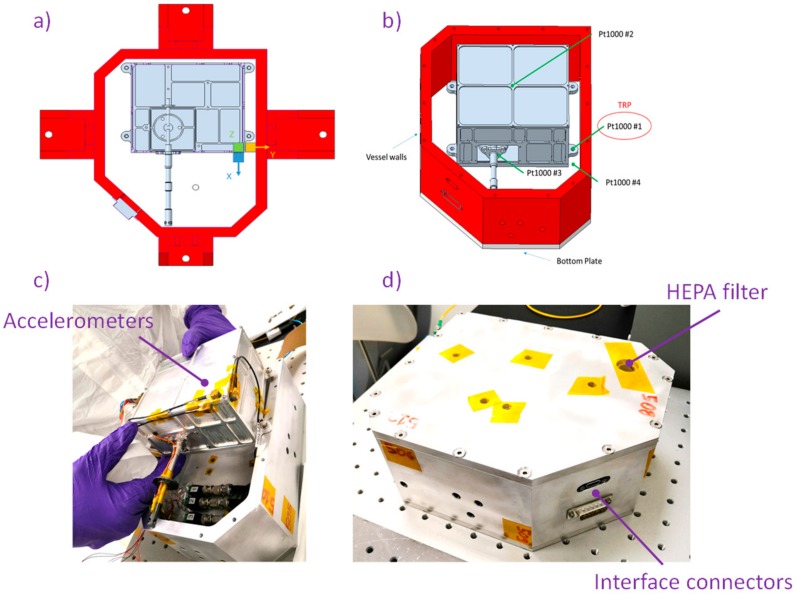
(**a**) Accelerometers positioning on the MicroMED; (**b**) temperature detectors mounting positions; (**c**) view of the MicroMED during integration within the vessel; (**d**) vessel after integration.

Views of the sensors positioning for mechanical and thermal tests and of the instrument during integration are shown in Figure 10. The box allows measurements of the acceleration, temperatures, and instrument data (temperatures, detectors’ output, PS performances) by means of interface connectors located on a wall of the container. 

Stability of instrument’s parameters, like the voltage of the reference detector and the PS rotational speed and power consumption, were identified as success criteria for the qualification testing.

## 4. Qualification Testing

### 4.1. Mechanical Testing

**Figure 11:**
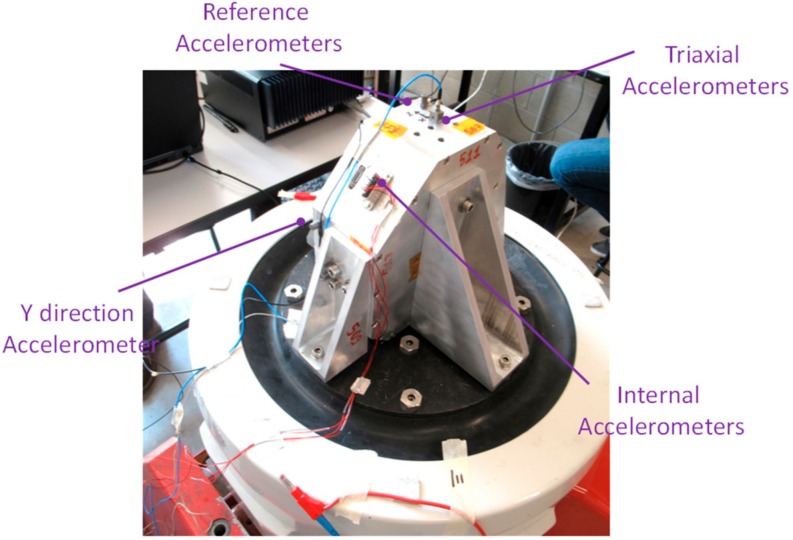
Testing unit mounting, qualification along X axis.

**Figure 12:**
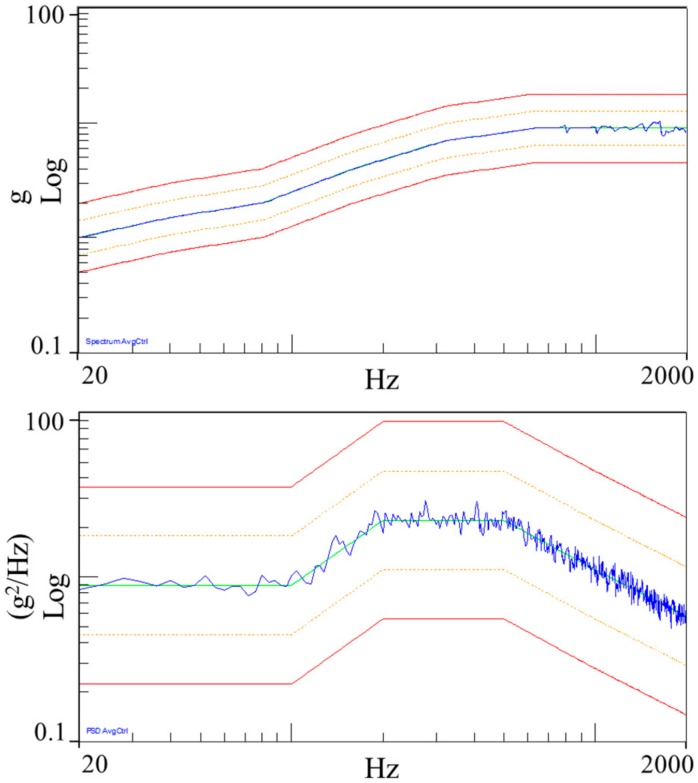
Applied sweep sine (top) and random (bottom) acceleration levels. Blue trend is the acceleration level measured at the interface whereas green trend shows reference profile. Control acceleration levels at 3 dB and 6 dB are shown in orange and red colors.

**Figure 13:**
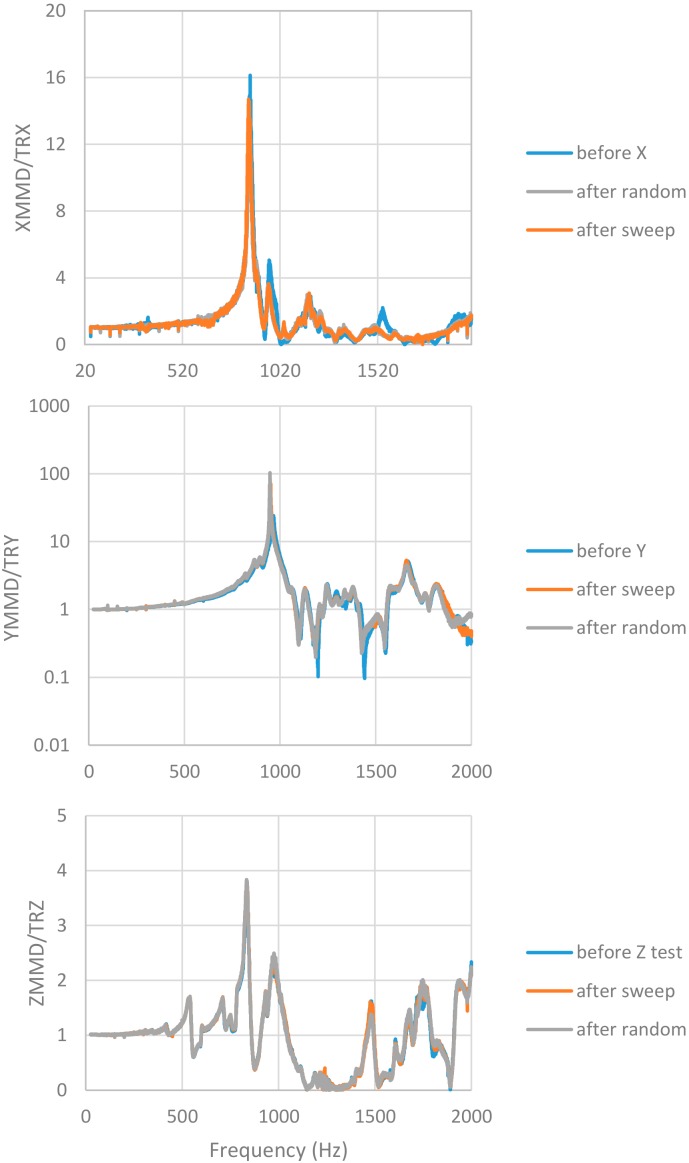
Measured frequency response functions (between internal accelerometer and reference one) for X (top), Y (middle), and Z (bottom) directions for the mechanical qualification.

Facility for mechanical testing is based on an LDS V830 SPA-16 electro-dynamic shaker, with LMS SCADAS III controller for sine, random, and shock excitation. The shaker provides 8.9 kN peak force, maximum sine acceleration of 734.5 m/s^2^, and nominal bandwidth up to 2250 Hz. The test procedure required testing of the unit along the three axes X, Y and Z, identified in accordance with the MicroMED mounting on the Dust Suite, with the acceleration levels and profiles summarized in Table 1 and Table 2. Before and after each power test (sweep sine or random), a resonance search test was performed. The latter was a sweep sine test at low acceleration amplitude (5 m/s^2^) from 20 Hz to 2000 Hz. This test measures, by means of installed accelerometers, the instrument resonances and tracks any change in the MicroMED dynamic behavior. In order to control applied acceleration at the mounting interface and monitor box accelerations, a triaxial (256B21 model, PCB Piezotronics,) and two monoaxial (4397 model, B&K) accelerometers were used. Accelerometers were calibrated with Brüel & Kjaer Calibration Exciter, type 4294. Figure 11 shows installation of the tested unit on the shaker for qualification along the X direction and related acceleration levels for power sweep and random are shown in Figure 12. Figure 13 provides measured frequency response functions during resonance search tests for the complete qualification.

### 4.2. Thermo-Vacuum Testing

**Figure 14:**
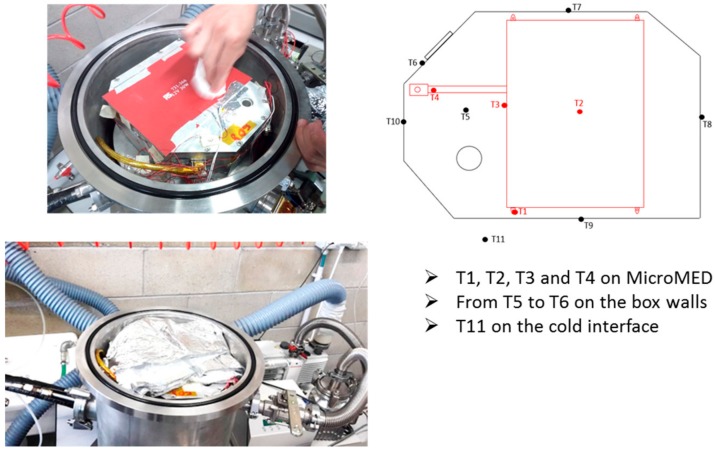
(Left) View of the mounting of the test unit inside the chamber and (right) scheme of the additional temperature sensors for the thermo-vacuum qualification.

Cylindrical thermo-vacuum (TV) chamber (300 mm in diameter and 400 mm in length) was used for qualification testing. Both ends of the cylinder are removable flanges and inside the chamber a LiN2 cooled plate is used as a heat sink. Film heaters were used for fine thermal control. The TV chamber comprises a pressure gauge in the range from 1 bar to 10^−4^ Pa (Cathode Gauge LIC40239 gauge, Varian), a CC2C Varian conditioning unit, and connection with 37972A Keysight conditioning unit for temperature reading. Varian DS 402 rotary vane pump and T-Station 85 turbomolecular pumping station were used to achieve the required low pressure level in the vacuum chamber. In addition to temperature sensors on MicroMED parts (read by the instrument electronics) and the four ones installed on the MicroMED during integration on the protection box, additional sensors (named from T5 to T10) were installed on the testing unit and cold interface (T11) of the chamber. Figure 14 provides views of the box installation inside the vacuum chamber and of the temperature sensors’ positioning.

Qualification requires temperature stabilization at defined test points (variation of less than 1 °C per hour) and its maintenance for at least 2 h dwell time. Thermo-vacuum levels and cycles for qualification are summarized in Table 6.

**Figure 15:**
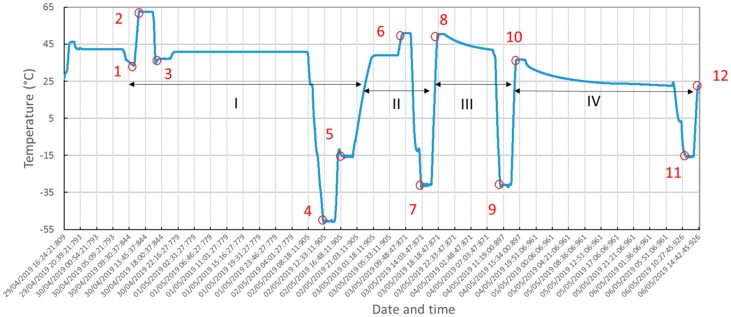
Thermo-vacuum cycles, measured temperature at the TRP.

Some check points were defined during vacuum testing (at safe temperatures for the instrument’s components) to monitor performance of the MicroMED (i.e., in particular the outputs of the detectors, straight light measurements and PS performances). Measured temperatures at the TRP are provided in Figure 15 as well as the indication of performed cycles.

**Figure 16:**
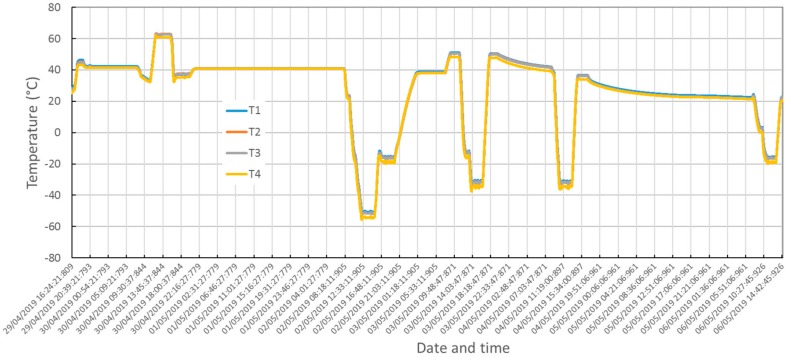
Thermo-vacuum qualification, measured temperatures, T1–T4 sensors.

**Figure 17:**
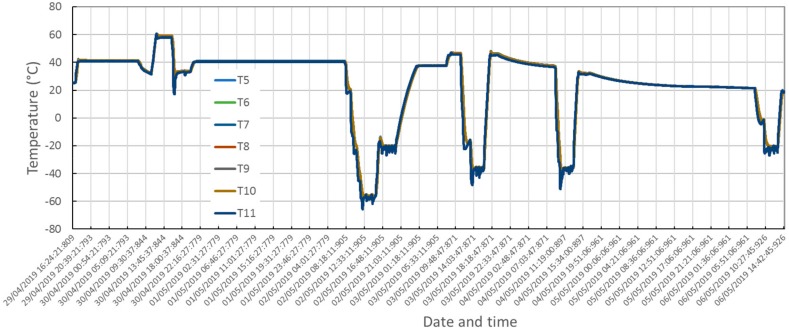
Thermo-vacuum qualification, measured temperatures, T5–T11 sensors.

Figure 16 shows overlap of the temperature readings from the sensors installed on the MicroMED during integration. Figure 17 shows measured temperatures from the additional sensors installed on the test unit.

### 4.3. Discussion

Qualification in mechanical environment of the MicroMED Flight Model was successfully completed. Mechanical testing evidenced survival of the instrument under expected mechanical loads. Functional tests performed after each direction did not show deviations from the nominal behavior of the instrument providing indirect proof that no mechanical changes occurred during the testing. Moreover, changes in measured resonances before and after power vibration tests (sine and random) were found to be within the allowed limits (less than 5 % of the reference value) justified by normal settlement of the mechanical structure bolted junctions.

Qualification in the thermal environment was successfully performed. No criticalities were detected during testing as shown by temperature plots in Figure 15, Figure 16 and Figure 17. The optical group and the LG survived storage at maximum and minimum temperature predicted for the mission’s scenarios. Moreover, checking the instrument’s performance and housekeeping during thermal cycling evidenced compatibility of the measured straight light levels and PS power consumption with the reference values before qualification, validating the thermo-mechanical design and the workmanship of integration activities.

The main result of the successful qualification was that MicroMED Flight Model was ready to be delivered to the Russian team of IKI (Space Research Institute of the Russian Academy of Sciences) for the integration activity on the Dust Suite package.

## 5. Conclusions

This work provides validation of the thermo-mechanical design of MicroMED, a miniaturized particle analyzer for the ExoMars 2020. Modal and quasi-static analyses performed with finite element model allowed verification of the dynamic behavior and mechanical strength of the instrument, required to be compliant with the expected mechanical and thermal scenarios. The thermo-elastic and thermal analyses provided the generated stresses and the expected temperature distribution in different operational and non-operational conditions, showing that no criticalities were foreseen for the most sensitive components (i.e., the laser group and the pumping system). Moreover, predicted thermo-elastic behavior demonstrated no critical conditions within the qualification temperature range, spanning from −50 °C to 60 °C. The MicroMED Proto-Flight Model was manufactured and integrated for qualification testing. Qualification campaign was successfully completed both in mechanical and thermal environments, proving MicroMED’s compliance with all conditions throughout all mission phases. Moreover, thanks to the stability of MicroMED’s measured performances during the qualification, compliance with the operative environments of the sensor’s optical and thermo-mechanical designs was demonstrated as well.

## Figures and Tables

**Table 1:** Power sweep sine excitation.

Frequency (Hz)	Level ^1^ (g)
20	1
40	1.5
80	2
160	4
320	7
640	9
2000	9

^1^ “g” has the meaning of standard acceleration gravity (i.e., about 9.81 m/s^2^).

**Table 2:** Random levels.

Frequency (Hz)	Level ^2^ (g^2^/Hz)
20	0.02
50	0.02
100	0.02
200	0.05
500	0.05
1000	0.025
2000	0.013

^2^ “g” has the meaning of standard acceleration gravity (i.e., about 9.81 m/s^2^).

**Table 3:** Mechanical and thermal properties of the materials.

Material	Density (kg/m^3^)	Modulus of Elasticity (MPa)	Poisson’s Coefficient	Tensile Yield Strength (MPa)	CTE (10^−5^/K)	Thermal Conductivity (W/(mK))
Al 7075	2810	71,700	0.33	503	2.36	130
Al 6061	2700	68,900	0.33	55.2	2.34	180
Vespel SP22	1650	4830	0.4	51.7	3.8	0.89

**Table 4:** Von Mises stresses for mechanical analyses.

Analysis	Maximum Von Mises Stress (MPa)	Margin of Safety (MOS)
Quasi-static X axis	129	0.62
Quasi-static Y axis	125	0.63
Quasi-static Z axis	207	0.38
Thermo-elastic cold case non-operative condition	248	0.26

**Table 5:** Thermo-optical properties ^3^.

Part	Material	Coating	$\alpha_{s}$	$\rho_{s}$	$\varepsilon_{h}$	$\rho_{h}$
MicroMED	Al 7075	Surtec 650	ns	ns	0.12	0.88
Electronics	PCB	PCB	ns	ns	0.8	0.2
OH	Al 7075	Aeroglaze Z306	0.95	0.05	0.9	0.1

^3^ “s” means specular, “h” means hemispherical, “ns” means not significant.

**Table 6:** Maximum and minimum temperatures for the qualification.

Cycle Number	Maximum Temperature (°C)	Minimum Temperature (°C)
I	60	−50
II	50	−30
III	50	−30
IV	35	−15
